# Genetic Diversity and Population Differentiation of a Chinese Endangered Plant *Ammopiptanthus nanus* (M. Pop.) Cheng f.

**DOI:** 10.3390/genes14051020

**Published:** 2023-04-29

**Authors:** Aoran Li, Miao Ma, Haotian Li, Songfeng He, Shugao Wang

**Affiliations:** Ministry of Education Key Laboratory of Xinjiang Phytomedicine Resource Utilization, College of Life Sciences, Shihezi University, Shihezi 832003, China

**Keywords:** *Ammopiptanthus nanus* (M. Pop.) Cheng f., endangered species, genetic differentiation, genetic diversity, inter-simple-sequence repeat (ISSR)

## Abstract

*Ammopiptanthus nanus* (M. Pop.) Cheng f. is a very important resource plant that integrates soil and water conservation, afforestation of barren mountains, and ornamental, medicinal, and scientific research functions and is also a critically endangered plant in China, remaining in only six small fragmented populations in the wild. These populations have been suffering from severe anthropomorphic disturbances, causing further losses in genetic diversity. However, its genetic diversity level and genetic differentiation degree among the fragmented populations are still unclear. Inthis study, DNA was extracted from fresh leaves from the remnant populations of *A. nanus*, and the inter-simple-sequence repeat (ISSR) molecular marker system was used to assess its level of genetic diversity and differentiation. The result was that its genetic diversity is low at both species and population levels, with only 51.70% and 26.84% polymorphic loci, respectively. The Akeqi population had the highest genetic diversity, whereas the Ohsalur and Xiaoerbulak populations had the lowest. There was significant genetic differentiation among the populations, and the value of the genetic differentiation coefficient (Gst) was as high as 0.73, while the gene flow value was as low as 0.19 owing to spatial fragmentation and a serious genetic exchange barrier among the populations. It is suggested that a nature reserve and germplasm banks should be established as soon as possible for elimination of the anthropomorphic disturbances, and mutual introductions between the populations and introduced patches of the species, such as with habitat corridors or stepping stones, should be performed simultaneously to improve the genetic diversity of the isolated populations for the conservation of this plant.

## 1. Introduction

Genetic diversity is the amount of genetic variability present among individuals of a variety or population within a species. It is the product of the recombination of genetic material (DNA) during the inheritance process, mutation, gene flow, and genetic drift [1], and it results in variations in DNA sequence, epigenetic profiles, protein structures and isoenzymes, physiological properties, and morphological properties [2].

Genetic diversity within a species is a determinant of its successful response to natural or anthropogenic disturbance events, and the level of genetic diversity also determines evolutionary trends in a species. High genetic diversity improves species’ adaptability [3]; thus, decreases in genetic diversity may reduce the adaptative potential of a species [4], typically resulting in a higher probability of extinction. Generally, it is believed that the genetic diversity of endangered plants is lower than that of ordinary species due to the small number of remaining plants, genetic drift caused by inbreeding or self-inbreeding, and an increase in homozygotes [5]. Studies on the genetic diversity of endangered species can reveal the mechanisms of their endangerment [6] and provide a scientific basis for conservation strategies [7]. Thus, this has gained increasing attention from conservation biologists [8,9].

Habitat fragmentation can transform a large and continuous population into several small, isolated parts surrounded by heterogeneous landscapes [10,11]. The level of inter-population gene flow decreases as isolation increases, leading to increased genetic differentiation among populations. Small populations tends to have low genetic diversity [12] and, thus, a high risk of local extinction [13,14]. Intra- and inter-population genetic variation, gene flow, and genetic differentiation between fragmented populations may provide further information on the causes of their endangerment and aid in formulating effective conservation strategies.

**Figure 1:**
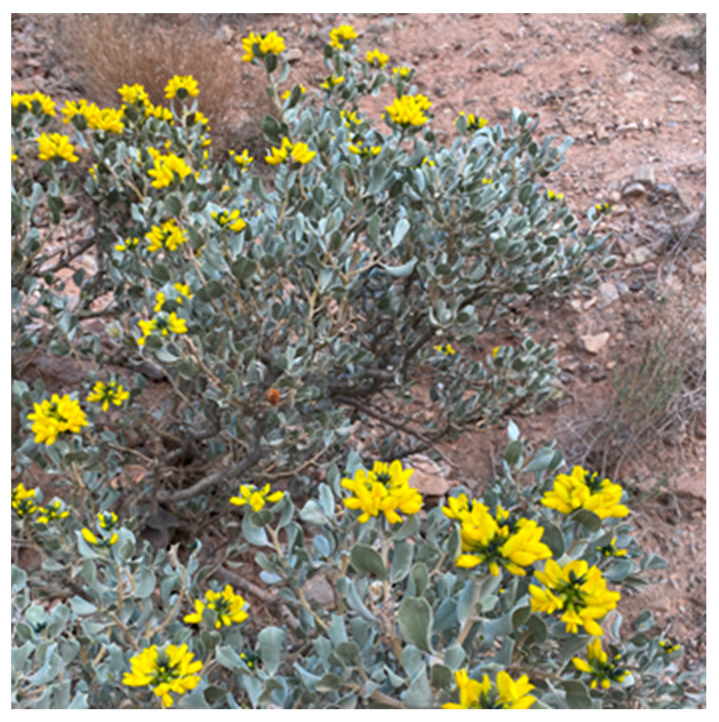
Flowers of *A. nanus*.

A very valuable relict plant, *A. nanus* is the Leguminaceae family and is a solo broad-leaved evergreen plant in the desert region of northwest China [15]. It is only found in a limited area in Kyrgyzstan [16] and in the Wuqia County of China, at an altitude of 2000–2400 m [15,17], growing on sunny arid slopes with 138 mm of average annual precipitation, 2580 mm of annual average evaporation, a 35 °C extreme maximum temperature, and a −30 °C of extreme minimum temperature, and is considered a super-arid plant with outstanding biological properties of drought tolerance [18] and cold tolerance [19,20,21] as well as the ecological values of wind protection, sand fixation, and prevention of soil erosion; its flower number is large, and its yellow corolla and “explosive” opening pattern in a short time has high ornamental value (Figure 1); its stems and leaves are rich in alkaloids, flavonoids, and phenylpropanoids [22], which were traditionally used for medicinal purposes; and it also has important academic value in the study of paleoclimatic changes.

However, climate change and long-term anthropogenic and natural disturbance events have resulted in a thin population of the species, with only six small, isolated surviving populations [23,24,25], and the species is listed as a national endangered plant in China. Furthermore, the species has since been strongly disturbed by grazing, logging, flooding, insect pests, and road-building in the past two decades [17]. Death is common in its wild populations; however, seedlings are extremely rare. Its genetic diversity has likely changed. For example, 31 individuals existed in the eastern Kangsu population 20 years ago, but only two currently remain due to highway-building, and the population is on the verge of extinction.

Although the level of genetic diversity and the population differentiation of the plant has been examined, there are great differences between the results of different studies. The results of Ge et al. (2005) showed that there was a low level of genetic differentiation, with only 8.5% of genetic differences occurring among populations [26]; however, Chen et al. [27] and Zhao et al.’s research [28], based on allozymes and RAPD molecular markers respectively, showed opposite results. Since a precise study on the degree of genetic differentiation and the level of genetic diversity is lacking, it is difficult to develop effective conservation measures for this endangered species.

There are many molecular markers available these days to analyze genetic diversity and population genetic structure in plants. The most frequently used molecular markers are inter-simple-sequence repeats (ISSR) [29]. The main advantages of using this method is that it is highly efficient, quick, inexpensive, robust, reproducible, highly polymorphic, randomly distributed throughout the genomes, and widely applicable to any genome [7]. The method has been effectively used for estimation of the genetic diversity and population genetic structure of plant resources [30,31,32].

Therefore, fresh leaves from all surviving populations of *A. nanus* were collected in this study, and inter-simple-sequence repeat (ISSR)-based genetic structure analysis was conducted for examining the genetic diversity level and genetic differentiation extent of the species and proposing effective conservation strategies.

## 2. Materials and Methods

According to the literature and our field investigations, there are only six populations of *A. nanus* (Tuopa, Akeqi, Kangsu, Xiaoerbulak, Ohsalur, and Heiziwei) surviving in Wuqia county in China (Table 1). Twenty individuals in each population were sampled randomly, and one young and healthy leaf of each individual was collected in 2022, regardless of size or age. The leaf was placed in a plastic self-sealing bag, and sufficient dried silica gel was added to rapidly dehydrate the leaves until DNA extraction.

DNA Extraction and PCR Amplification: DNA was extracted from the leaves using the DNA secure New Plant Genomic DNA Extraction Kit DP-3200 (Beijing Tiangen Biochemical Technology Co., Ltd. Germany). The extracted DNA was checked for purity and quality by agarose gel electrophoresis and was stored at −80 °C.

Polymerase chain reaction (PCR) amplification was performed using a Mastercycler Nexus X2 (Germany). The total volume of the amplification system was 25 μL, containing 2 μL of template DNA, 2 μL of primers, 12.5 μL of 2 × *Taq* PCR Mix, and 8.5 μL of ddH_2_O. Initial denaturation was performed at 94 °C for 5 min, followed by 45 cycles of 30 s at 94 °C, 60 s at 50–54 °C (depending on the primer), and 120 s at 72 °C; the final extension was at 72 °C for 7 min.

Nuclear DNA was amplifified by PCR using ISSR primers from the University of British Columbia primer set 9 (University of British Columbia, primer set #9). Following an initial screening from 100 primers, 10 primers that obtained maximum numbers of reliable and reproducible polymorphisms were then selected for analysis of the populations (Table 2).

Only bands that were clearly recorded across all populations were used. The ISSR profiles for each sample were scored on the presence or absence of the amplified products. The percentage of polymorphic loci (P), the average number of observed alleles per locus (N_A_), the effective number of alleles per locus (N_E_), Shannon’s information index (I), Nei’s gene diversity index (H), the genetic differentiation coefficient (Gst = 1-Hs/Ht, where Ht is the overall genetic diversity and Hs is the genetic diversity within a population), gene flow level (Nm), genetic distance, and genetic similarity were calculated using the POPGENE 32 software. SPSS 20.0 software was used for statistical analysis of the genetic diversity of *A. nanus* at species and population levels, and Duncan multiple comparisons were used for significant difference tests of N_A_, N_E_, H, and I among the populations (*p* < 0.05). The data are presented in the form of mean ± standard error. UPGMA clustering maps were constructed using NTSYS 2.1 software.

## 3. Results

### 3.1. Genetic Diversity Analysis

In this study, the 10 ISSR primers produced 136 unambiguous electrophoretic bands, among which 70 loci were polymorphic. The genetic diversity of *A. nanus* was low, with 51.70% and 26.84% polymorphic bands (P) at the species and population levels, respectively. However, the diversity parameters differed significantly among the six populations (Table 3).

### 3.2. Gene Flow

Our results show that the Nm of *A. nanus* is only 0.19, which is a very low level (Table 4).

### 3.3. Genetic Distance and Genetic Similarity among Populations

The genetic distances among the populations were relatively short, from 0.22 to 0.58, with a mean of 0.42. The populations showed a moderate level of genetic similarity (0.56–0.80), with a mean value of 0.66. The genetic similarity between the Ohsalur and Akeqi populations was the highest, but that between Ohsalur and Tuopa populations was the lowest (Table 5).

### 3.4. UPGMA Clustering

**Figure 2:**
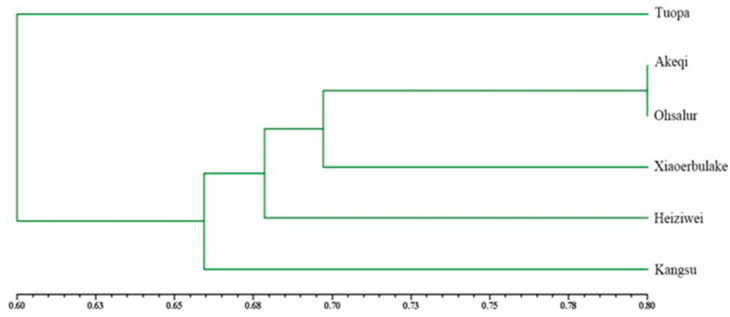
UPGMA clustering between populations of *A. nanus* based on genetic distance.

An UPGMA clustering map of the populations was constructed using NTSYS 2.1 software (Figure 2), which showed that the genetic distance between Akeqi and Ohsalur populations was the shortest.

## 4. Discussion

Study of genetic diversity and the population differentiation of an endangered species is a basis for exploring the adaptation of the species to its environment and is core to conservation biology. The genetic diversity of plant populations is determined by many factors, such as habitat type, reproductive mode, genetic mutation, genetic drift, and gene flow [33]. The percentage of polymorphic loci (P), Nei’s gene diversity index (H), and Shannon’s information index (I) are usually used for describing the genetic diversity level of a population or a species [26,34,35,36].

The genetic diversity revealed in our study is lower than that detected by Zhao et al. [28] and is much lower than that of its congener *A. mongolicus* [26]. The H and I of *A. nanus* were also extremely low at both species and population levels in the present study.

Zhao et al. (2016) found that the Kangsu population had the highest genetic diversity, the Xiaoerbulak population had the lowest, and the Ohsalur population was moderately diverse [28]. However, our results showed that, among the six populations, the Ohsalur and Xiaoerbulak populations had the lowest genetic diversity, with P values of less than 20% and H values of less than 0.1. Additionally, the P and NA of the Xiaoerbulak population were the lowest (16.91% and 1.17, respectively), and the NE, H, and I of the Ohsalur population were just 1.09, 0.06, and 0.09, respectively. Moreover, the Kangsu population had a moderate level of genetic diversity. These differences between the two works may be due to the individuals sampled, the primer sequences and molecular markers selected being different from each other, or the deaths of many individuals in Kangsu and Ohsalur populations because of the building of roads and construction of flood-control channels and reservoirs during the last decades, leading to an obvious reduction in population genetic diversity. Relatively speaking, the Akeqi population was the most genetically diverse, with the highest values for each parameter (P = 43.38, N_A_ = 1.43, N_E_ = 1.26, H = 1.53, and I = 0.23).

**Figure 3:**
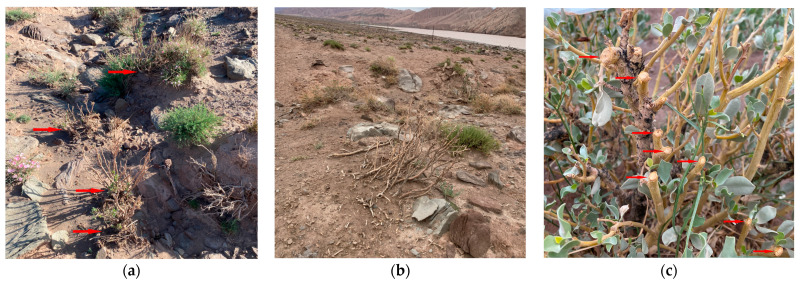

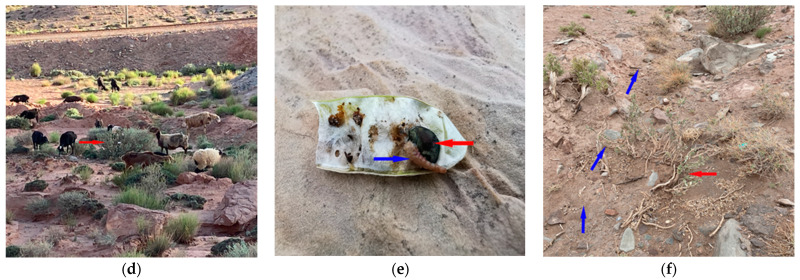
*A. nanus* at its habitat. (**a**) Effect of flooding; *A. nanus* is indicated by the red arrow. (**b**) Destroyed plants. (**c**) Effect of logging; the incision is indicated by red arrow. (**d**) Effect of grazing; *A. nanus* is indicated by the red arrow. (**e**) Effect of insect feeding; the seed of *A. nanus* is indicated by the red arrow and the insect is indicated by the blue arrow. (**f**) Broken by rocks; *A. nanus* is indicated by the red arrow and the blue arrow shows the gully.

Frequent disturbance events, such as flooding, (Figure 3a), road-building, (Figure 3b), logging, (Figure 3c), grazing, (Figure 3d), and insect feeding (Figure 3e) have severely reduced the fitness of the endangered plant. Although it was classified as a national first-class protected plant in China, no forms of in situ conservation measures have been taken, such as any level of nature reserve, and illegal felling still occurs in the field. During our field survey in 2021, we found that more than 20 individuals in the Ohsalur population were surreptitiously felled, and grazing was common in all populations [37]. Camels, horse, sheep, and goats are common livestock in the *A. nanus* communities, and camels and goats are the main herbivores and they prefer the buds, shoot tips, young branches, and leaves. Although *A. nanus* is a shrub, its branches are fragile; individuals along gullies are susceptible to damage from rocks rolling down along the mountain slope owing to flooding, and approximately 80% of individuals along gullies were broken by rocks (Figure 3f).

The overall Gst of the *A. nanus* populations was 0.73 (Table 4), indicating that 73% of the genetic variation existed among populations, and the genetic variation within each population was only 27%, which is consistent with the allelic enzyme analysis result from Chen et al. [26] and the RAPD analysis result from Zhao et al. [28], illustrating that the genetic similarity among the individuals within the population is high, the genetic differenations between the populations are severe, and there are some obvious inter-population gene flow obstacles.

Govindaraju classified Nm, which indicates genetic exchange between populations, into three levels: high for Nm ≥ 1.00, moderate for 0.25 < Nm < 0.99, and low for Nm ≤ 0.25 [38]. Gene flow can prevent or reduce genetic differences between populations accumulated by isolation, and frequent gene flow (Nm > 1.0) can prevent differentiation between populations caused by genetic drift. At Nm < 1.0, genetic drift is the dominant cause of differentiation among populations [34,36,39,40]. Our results show that the gene-exchange level among the six wild populations of *A. nanus* is very low, which intensifies their inter-population genetic differentiation, and there is a clear trend of increase.

A complex breeding system was found in *A. nanus*, with both apomixis and sexual reproduction, and yields 30% and 28% fruit sets by spontaneous self-pollination and heterozygous pollination, respectively [41]. It has an explosive flowering pattern, with tens to hundreds of open flowers on each individual during its brief flowering period. This blooming pattern and its self-compatible mating system may increase the chance of self-pollination, which may reduce the genetic diversity of the offspring, producing a high level of genetic similarity within a population. *Anthophora* (*Dasymegilla*) *waltoni* Cockerell, *Megachile* (*Chalcodoma*) sp., and *Halictus* sp. are its main pollinators [42]; their effective pollination distance generally does not exceed 2.5 km. However, the six populations were strongly fragmented and separated by 30 km at least. Thus, lack of pollinators may contribute to the low Nm among the six populations.

Wuqia County is the historical refuge of the Tertiary glacial period relic species *A. nanus*. The present populations likely evolved from a few individuals that survived a previous bottleneck event; thus, genetic similarity among individuals within the present population is very high, owing to a founder effect [43].

An UPGMA clustering map showed that the genetic distance between Akeqi and Ohsalur populations was the shortest, although the spatial distance between the two sites was the greatest. Previous studies showed that the genetic distance between populations was proportional to the spatial distance between them [34,36,39]; however, inconsistencies also existed in this relationship [35,40]. The Akeqi and Ohsalur populations are spatially remote but genetically close; thus, the two populations are genetically similar, possibly owing to genetically similar ancestors. Since the genetic structure of the Tuopa poipulation was unique, and the genetic similarity between the Tuopa and Akeqi or Ohsalur populations was the lowest, propagules from Tuopa may be transplanted into other populations to improve their genetic diversity levels.

## 5. Conclusions

Populations of *A. nanus* face serious threats from low genetic diversity, isolation, and lack of gene flow, resulting in strong inter-population genetic differentiation. These populations have been suffering from severe anthropomorphic disturbances, causing further losses in genetic diversity. Survival of the endangered plant has been negatively affected by some anthropomorphic interference events, reducing the fitness of the species in its native habitat. The population size has been decreasing and is rarely replenished by seedlings. Thus, genetic diversity of the species will continue to decrease if no positive steps are taken.

Therefore, a nature reserve for this species and germplasm banks should be established as soon as possible, and anthropomorphic disturbances, such as felling, road-building, or grazing activities, should be strictly prohibited. Second, manual interventions through seed sowing and mutual transplantation among populations should be carried out to increase populations’ genetic diversity. In particular, since the Akeqi and Heiziwei populations are highly genetically diverse, seeds can be collected from these populations and widely sown in other populations to improve their genetic diversity.

Identifying species distribution and habitat change is an important part of effective conservation management. Nevertheless, economic development and rapid population growth worldwide in the past few decades have led to dramatic changes in land cover, habitat fragmentation and loss of species, especially in China [44,45]. Habitat fragmentation is commonly brought on by the human activities of logging, building roads, and other construction. When large areas of habitat are fragmented, resources are reduced, and this can lead to declines in a species’ population and even threaten its survival. Fragmentation divides individuals into several parts within a population and cuts them off from crucial resources [46]. Habitat fragmentation and loss can also reduce population connectivity between habitat patches, increase isolation, alter the composition and configuration of key habitats, increase the risk of species extinction, and ultimately affect biodiversity and ecosystem health. A dense population and a developed economy characteristic of this region would lead to high consumption of natural resources. Therefore, coordinating biodiversity conservation and economic development at a minimum cost has become a complex problem for stakeholders and scientists in conservation planning in China.

Landscape connectivity has potential effects on the survival, fitness, gene flow, diversity, and colonization of distinct small populations and has changed habitat suitability in the area. Habitat corridors save limited land resources and are the main countermeasure for species to reduce the negative impacts of habitat fragmentation and loss, which can promote the connectivity of habitat patches, increase habitat connectivity, and maintain material, energy, and gene flows and interactions among populations in remaining habitat patches [47]. The ecological network approach aims to expand the integrity of environmental processes, facilitate the conservation of species and habitats, and promote biodiversity, and a stepping stone is a specific kind of corridor where small patches provide habitats for shelter, feeding, or resting [48].

Due to the long-term impact of human activities, *A. nanus*’s habitats are facing the threat of fragmentation and loss, posing a serious challenge to its survival and diversity. Introduced patches of *A. nanus* should be planted in suitable ranges between the six surviving populations as habitat corridors or stepping stones to reduce their degree of isolation and increase gene flow among them; thereby, the adaptability potential of this species may be improved.

The reasons for the endangerment of this plant are multifaceted, among which pests in nature are one of the important reasons. Due to the harsh environment of *A. nanus*, there are few plant species available for insects to feed on, and the pest damage faced by the endangered plant is becoming increasingly severe. During our continuous years of investigation, we found that the insect infestation rate of seeds in natural populations is as high as 90%; rampant pest infestation is one of the main factors causing low seed yield and population renewal barriers for this plant. *Etiella zinckenell* is the pest that poses the most serious threat to the seeds of *A. nanus*, so it is necessary and urgent to conduct studies on prevention and control of this pest in the future, making sure its population density can be controlled at a low level to avoid a major outbreak of this pest.

## Figures and Tables

**Table 1:** Sampling locations for *A. nanus*.

Collection Site	Longitude	Latitude	Altitude (m)	Number of Samples
Tuopa	75°34′54.62″	39°46′26.11″	2018	20
Akeqi	75°35′24.26″	39°49′54.45″	2059	20
Heiziwei	75°18′58.45″	39°48′9.34″	2402	20
Kangsu	75°03′22.00″	39°42′12.00″	2161	20
Ohsalur	74°45′18.00″	39°39′30.00″	2202	20
Xiaoerbulak	75°00′53.41″	39°41′47.35″	2217	20

**Table 2:** Primer information.

Primers	Strios Number	Polymorphic Band	Percentage of Polymorphic Bands	Primer Sequences (From 5′ End to 3′ End)	Fire Retardant Temperature
UBC807	16	9	56.25%	AGA GAG AGA GAG AGA GT	53 °C
UBC810	15	8	53.33%	GAG AGA GAG AGA GAG AT	50 °C
UBC811	16	10	62.50%	GAG AGA GAG AGA GAG AC	45 °C
UBC822	6	3	50.00%	TCT CTC TCT CTC TCT CA	50 °C
UBC834	13	7	53.85%	AGAGAGAGAGAGAGAGYT	49 °C
UBC835	13	6	46.15%	AGAGAGAGAGAGAGAGYC	52 °C
UBC840	14	8	57.14%	GAGAGA GAG AGA GAG AYT	50 °C
UBC844	15	8	53.33%	CTC TCT CTC TCT CTC TRC	49 °C
UBC888	14	5	35.71%	BDB CAC ACA CAC ACA CA	41 °C
UBC891	14	6	42.86%	HVH TGT GTG TGT GTG TG	55 °C
Total	136	70	51.47%		

Note: B = (C, G, T) (i.e., not A), D = (A, G, T) (i.e., not C), H = (A, C, T) (i.e., not G), Y = (C, T).

**Table 3:** Genetic diversity of *A. nanus* at the species and population levels.

Population/Species		P (%)	N_A_	N_E_	H	I
Population	Tuopa	22.79	1.23 ± 0.42 ab	1.16 ± 0.32 a	0.09 ± 0.17 ab	0.13 ± 0.25 ab
	Akeqi	43.38	1.43 ± 0.50 b	1.26 ± 0.35 b	0.15 ± 0.20 c	0.23 ± 0.28 c
	Heiziwei	31.62	1.32 ± 0.47 b	1.17 ± 0.29 a	0.11 ± 0.17 b	0.16 ± 0.25 b
	Kangsu	28.68	1.29 ± 0.45 b	1.16 ± 0.30 a	0.09 ± 0.17 ab	0.14 ± 0.24 ab
	Ohsalur	17.65	1.18 ± 0.38 a	1.10 ± 0.24 a	0.06 ± 0.14 a	0.09 ± 0.21 a
	Xiaoerbulak	16.91	1.17 ± 0.38 a	1.12 ± 0.30 a	0.07 ± 0.16 ab	0.10 ± 0.23 ab
	Mean	26.84	1.27	1.16	0.10	0.14
Species		51.70	1.99	1.60	0.35	0.52

Note: P: percentage of polymorphic loci; N_A_: observed number of alleles; NE: effective number of alleles; H: Nei’s gene diversity index; I: Shannon’s information index. The different lowercase letters mean there are significant differences among the populations.

**Table 4:** Genetic differentiation among populations.

Ht	Hs	Gst	Nm
0.35 ± 0.02	0.10 ± 0.01	0.73	0.19

Note: Ht: overall genetic diversity; Hs: genetic diversity within a population; Gst: genetic differentiation coefficient among populations; Nm: gene flow.

**Table 5:** Nei’s genetic similarity and the genetic distances of the six populations.

Populations	Tuopa	Akeqi	Heiziwei	Kangsu	Ohsalur	Xiaoerbulak
Tuopa	******	0.610	0.622	0.613	0.561	0.618
Akeqi	0.495	******	0.669	0.674	0.804	0.729
Heiziwei	0.475	0.402	******	0.647	0.681	0.701
Kangsu	0.489	0.395	0.435	******	0.672	0.663
Ohsalur	0.578	0.218	0.385	0.397	******	0.675
Xiaoerbulak	0.481	0.316	0.356	0.411	0.393	******

Note: Genetic similarity is above the diagonal, and genetic distance is below the diagonal, ****** representing the demarcation line.

## Data Availability

Not applicable.
